# Case Report: Diagnostic overshadowing in a patient with major depressive symptoms later diagnosed with systemic amyloidosis

**DOI:** 10.3389/fpsyt.2026.1906103

**Published:** 2026-08-12

**Authors:** Yuexi Liang, Wei Li, Lanying Liu

**Affiliations:** 1 Shanghai Mental Health Center, Shanghai Jiao Tong University School of Medicine, Shanghai, China; 2 Shanghai Institute of Traditional Chinese Medicine for Mental Disorders, Shanghai, China

**Keywords:** depression, diagnostic overshadowing, neurovascular dysfunction, psychosomatic interaction, systemic amyloidosis

## Abstract

**Background:**

The symptoms of depression may overlap with the early manifestations of systemic diseases, resulting in diagnostic masking. Amyloidosis is a multisystemic disease that has the potential to affect the heart, kidneys, nervous system, liver, lungs and intestines. Cardiac involvement is the leading cause of morbidity and mortality. As the symptoms of systemic amyloidosis can be non-specific and like those of a depressive episode in its early stages, there can be delays in diagnosis when patients are initially diagnosed depressed.

**Case presentation:**

We report the case of a 57-year-old male patient who was initially diagnosed with a major depressive episode. During treatment, he developed recurrent episodes of hypotension and syncope, which were initially attributed to his psychiatric condition and medication-related factors. As his clinical condition progressively deteriorated, a renal biopsy was performed, which ultimately confirmed a diagnosis of systemic amyloidosis with cardiac involvement and acute decompensated heart failure.

**Conclusion:**

This case suggests that, at the interface between psychiatry and somatic medicine, diagnostic overshadowing, anchoring bias, and fragmented care may jointly contribute to delayed recognition of systemic amyloidosis, particularly cardiac amyloidosis. In cases where patients present with depression-like manifestations, orthostatic hypotension, and nonspecific multisystem symptoms, clinicians should remain highly alert to persistently progressive physiological abnormalities and dynamically re-evaluate potential underlying somatic etiologies as new evidence emerges. Greater interdisciplinary awareness and reduced cognitive bias may help to reduce diagnostic delay and optimize multidisciplinary management. Further investigation is required to explain the mechanistic links between systemic amyloidosis and depressive symptoms.

## 1 Introduction

Depression is a highly prevalent condition among patients with cardiovascular disease (CVD), diabetes, chronic obstructive pulmonary disease (COPD) and severe asthma. It is one of the most common comorbidities in people with more than one disease (multi-morbidity) (1). When comprehensive diagnostic information is lacking, diagnostic overshadowing occurs when mental health professionals attribute physical symptoms reported by mental health service users to psychiatric conditions (2).

Systemic amyloidosis is a group of diseases characterized by extracellular deposition of misfolded fibrillar proteins across multiple tissues. The incidence of immunoglobulin light chain (AL) amyloidosis is 12 cases per million persons per year. Wild-type ATTR (amyloid transthyretin) is estimated to have a prevalence 155 to 191 cases per million persons. The incidence of variant ATTR amyloidosis is estimated at 0.3 cases per year per million persons, with a prevalence estimate of 5.2 cases per million persons. Systemic amyloidosis results in a high symptom burden, impairment of quality of life, and a shortened survival (3).

Cardiac involvement has been identified as the primary cause of morbidity and mortality in cases of amyloidosis (4). Cardiac amyloidosis (CA) denotes myocardial involvement resulting from extracellular space infiltration by amyloid deposits (5). Among amyloid subtypes, immunoglobulin light chain cardiac amyloidosis (AL-CA) and transthyretin cardiac amyloidosis (ATTR-CA) most commonly cause infiltrative cardiomyopathy (6). The initial clinical presentation of CA depends on its underlying subtype (7). In AL amyloidosis, nearly all organs may be affected beyond the central nervous system, with common manifestations including exertional dyspnea, reduced exercise tolerance, fatigue, and peripheral oedema (8). The clinical presentation of ATTR-CA is highly variable and depends on the specific genotype. Carpal tunnel syndrome presents as the initial symptom in 33% of patients, subsequently leading to peripheral neuropathy, autonomic neuropathy, and cardiac involvement. Autonomic dysfunction manifests as polyneuropathy, causing urogenital, gastrointestinal, or cardiac circulatory symptoms. These include nausea, vomiting, constipation, orthostatic hypotension, urinary retention, and sexual dysfunction (9). Diagnosing CA is often challenging, as early signs and symptoms may be non-specific.

We present a case illustrating the evolution of clinical reasoning under conditions of incomplete information, and how cognitive biases and fragmented care can delay recognition of systemic amyloidosis presenting with prominent depressive symptoms.

## 2 Case presentation

A 57-year-old male patient presented to our psychiatric outpatient clinic in June 2025 with a three-month history of sleep disturbance and progressive somatic complaints.

His symptoms commenced shortly after attending social celebrations during the Spring Festival period. By March, he had trouble initiating sleep, primarily characterized by generalized aching and an inability to lie flat. He reported low mood during the day but maintained occupational functioning.

Over subsequent weeks, the patient experienced progressive loss of appetite, describing food as “tasteless”. Between March and May, his weight decreased from approximately 80 kilograms to 60 kilograms. Gastrointestinal endoscopy revealed no structural abnormalities, and routine outpatient investigations (specific details not provided) also reported no significant abnormalities.

The patient reported persistent depressed mood, loss of pleasure, and decreased energy, together with sleep disturbance, appetite changes, and weight loss. In the absence of psychotic symptoms, the condition was diagnosed as a severe depressive episode without psychotic symptoms according to ICD-10 criteria (F32.2). Treatment commenced with duloxetine and low-dose olanzapine.

Olanzapine was maintained at 5 mg daily, whilst duloxetine was titrated to 60 mg according to standard protocols. However, no significant improvement in mood symptoms was observed. Fatigue worsened, and recurrent daytime falls commenced. During these episodes, recorded blood pressure readings dropped to a minimum of 70/40 mmHg.

Given olanzapine’s association with orthostatic hypotension, this medication was discontinued. Nevertheless, hypotensive symptoms showed no marked improvement post-discontinuation. During August, the patient experienced syncopal episodes.

Emergency assessment reportedly revealed abnormal cardiac biomarkers and elevated brain natriuretic peptide levels, suggesting potential cardiac dysfunction. However, transthoracic echocardiography demonstrated preserved left ventricular ejection fraction with no significant structural abnormalities. Treatment with duloxetine was continued.

In September 2025, the patient developed progressive dysphagia, tolerating only liquid or semi-liquid foods, with solid food intake triggering vomiting. The patient also reported severe sacrococcygeal pain, intense enough to awaken them from sleep.

Given recurrent syncope and persistent hypotension, further medical evaluation was recommended. However, endocrinological investigations revealed no significant abnormalities.

The patient revisited an external hospital in November for a renal biopsy, which demonstrated amyloid deposition consistent with renal amyloidosis. The current international guidelines recommend clone-directed therapy as the cornerstone of treatment for AL amyloidosis. Daratumumab-based regimens (Dara-CyBorD) are now considered the first-line therapy of choice, as evidence has demonstrated improved hematological response rates and recovery of organ function compared with conventional chemotherapy approaches (10). However, following the initiation of anti-plasmacytoma therapy involving daratumumab in combination with bortezomib, cyclophosphamide and dexamethasone, the patient developed symptoms of progressive heart failure.

In December, the patient was admitted to cardiology with a diagnosis of acute decompensated heart failure (New York Heart Association class IV) with preserved ejection fraction, secondary to primary systemic amyloidosis with cardiac involvement. Transthoracic echocardiography demonstrated concentric left ventricular hypertrophy (interventricular septum and posterior wall thickness of 15–19 mm), biatrial enlargement, preserved left ventricular ejection fraction (60%), and diastolic dysfunction. Advanced diagnostic modalities, including cardiac MRI, global longitudinal strain analysis, and nuclear scintigraphy, were not performed due to the patient’s critical clinical condition and acute decompensated heart failure, which limited further evaluation.

During hospitalization, the patient received vasopressor support (including noradrenaline and midodrine), diuretics (bumetanide and tolvaptan), and positive inotropic therapy (levosimendan) to improve cardiac output. Following stabilization of symptoms, the patient was discharged in late December.

At one-month follow-up, the patient continued diuretic therapy (furosemide), pressor support (midodrine), ivabradine, and spironolactone for heart failure management. The duloxetine dose had been reduced to 40 mg daily. His mood symptoms showed marked improvement, and gradual tapering of antidepressant medication is currently being considered. The patient’s entire diagnostic and treatment course is summarized in **Table 1**.

**Table 1:** Clinical timeline of the episode of care.

Date/period	Clinical manifestations	Diagnostic assessment/key findings	Management
June 2025 Initial psychiatric outpatient visit	3-month history of insomnia, generalized body pain, inability to lie flat, depressed mood, anhedonia, reduced appetite, and approximately 20 kg weight loss.	Gastrointestinal endoscopy showed no structural abnormalities. Routine outpatient evaluations reportedly showed no significant findings.	Symptoms met ICD-10 criteria for a severe depressive episode without psychotic symptoms (F32.2). Duloxetine and low-dose olanzapine were initiated.
After initiation of psychiatric treatment	Affective symptoms did not improve significantly. Fatigue worsened, and the patient developed recurrent falls with the lowest recorded blood pressure of 70/40 mmHg.	The temporal association with antipsychotic treatment raised concern for medication-related orthostatic hypotension.	Olanzapine was discontinued. Persistent hypotension after withdrawal weakened a purely iatrogenic explanation.
Subsequent outpatient/emergency evaluations	Hypotension persisted and syncopal episodes occurred.	Cardiac dysfunction and endocrine causes were considered. Emergency evaluation showed elevated BNP, whereas transthoracic echocardiography showed preserved left ventricular ejection fraction and no significant structural abnormality. Endocrinological investigations were unremarkable.	The initial depressive diagnosis was not immediately revised, and duloxetine was continued. Ongoing unexplained physiological abnormalities created uncertainty in management.
September 2025	The patient developed progressive dysphagia, tolerating only liquid or semi-liquid food; solid food intake triggered vomiting. He also reported severe sacrococcygeal pain that awakened him from sleep.	The emergence of gastrointestinal, pain-related, and autonomic/circulatory symptoms suggested a broader multisystem process.	Further somatic evaluation became clinically necessary.
November 2025	The patient revisited an external hospital for further evaluation.	Renal biopsy demonstrated amyloid deposition consistent with renal amyloidosis.	Anti-plasmacytoma therapy was initiated with daratumumab, bortezomib, cyclophosphamide, and dexamethasone. Progressive heart failure symptoms subsequently developed.
December 2025 Cardiology admission	The patient was admitted with progressive heart failure symptoms.	The patient was diagnosed with acute decompensated heart failure with preserved ejection fraction, New York Heart Association class IV, secondary to primary systemic amyloidosis with cardiac involvement.	Treatment included vasopressor support, diuretics, and positive inotropic therapy to improve cardiac output.
Late December 2025 Discharge	Symptoms stabilized after inpatient management.	The patient was discharged after stabilization. No long-term follow-up data.	The case highlights the need for dynamic diagnostic reassessment when depressive symptoms coexist with evolving multisystem abnormalities.

## 3 Discussion

### 3.1 Diagnostic reconstruction amid clinical uncertainty

The diagnostic trajectory in this case vividly illustrates how clinical hypotheses dynamically evolve in response to emerging information. At initial presentation, the patient primarily presented with insomnia, low mood, anhedonia, reduced appetite, and significant weight loss. The diagnosis of major depressive episode was clinically reasonable, given the absence of marked cardiopulmonary abnormalities and unremarkable gastrointestinal investigations. This symptom constellation met established diagnostic criteria for depression, with no warning signs suggestive of systemic disease. At this stage, the diagnostic probability favored a primary psychiatric disorder.

Subsequent recurrent falls accompanied by severe hypotension added complexity to management. Given the concomitant use of olanzapine and duloxetine, drug-induced orthostatic hypotension presented a plausible and straightforward explanation. Discontinuation of olanzapine thus constituted a reasonable therapeutic option. It is worth noting that no orthostatic blood pressure measurements were performed at that time, and the assessment was based on a clinical and empirical judgment rather than formal hemodynamic evaluation. However, the persistence of hypotension following this adjustment, accompanied by syncope, undermined the hypothesis of a purely iatrogenic mechanism. Diagnostic focus shifted from drug side effects towards potential systemic pathophysiology (**Table 2**).

**Table 2:** Serial measurements of NT-proBNP and high-sensitivity cardiac troponin I levels.

Laboratory results	13 Dec 2025	14 Dec 2025	15 Dec 2025	17 Dec 2025
NT-proBNP (pg/mL)	31006	18812	—	16892
hs-cTnI (pg/mL)	158.7	—	164.8	—

“—” = data not available at that time; NT-proBNP, N-terminal pro-B-type natriuretic peptide; hs-cTnI, high-sensitivity cardiac troponin I.

Emergency investigations further revealed abnormal cardiac biomarkers and elevated natriuretic peptide levels, suggesting cardiac overload and the potential for hypotension due to impaired left ventricular ejection capacity. However, echocardiography demonstrated preserved left ventricular ejection fraction, ruling out this possibility. The coexistence of biochemical evidence for cardiac overload with preserved systolic function necessitated re-evaluation of the diagnostic framework. Alternative mechanisms, such as restrictive cardiomyopathy or infiltrative cardiomyopathy, gained increasing plausibility relative to conventional systolic heart failure.

With the emergence of additional symptoms—including progressive dysphagia and persistent hypotension—the clinical presentation extended beyond isolated emotional disturbance or transient cardiovascular instability. Cumulative evidence of multisystem involvement prompted further investigation. Renal biopsy ultimately confirmed systemic amyloidosis, with subsequent cardiac decompensation validating cardiac involvement (**Table 3**).

**Table 3:** Serial measurements of serum light-chain-related biomarkers.

Laboratory results	13 Dec 2025	16 Dec 2025	Reference range
Kappa/lambda light chain ratio	1.83	—	0.26–1.65
Free kappa/lambda light chain ratio	0.71	0.81	0.26–1.65
Free kappa light chain (mg/L)	6.96	8.63	3.3–19.4
Free lambda light chain (mg/L)	9.85	10.7	5.7–26.3
Kappa light chain (g/L)	0.75	—	0.33–1.94
Lambda light chain (g/L)	0.41	—	0.57–2.63

“—” = data not available at that time.

Reviewing this case, multiple early clinical features—including orthostatic hypotension, recurrent syncope, preserved ejection fraction against a background of elevated cardiac biomarkers, and gastrointestinal dysfunction—were consistent with autonomic dysfunction and restrictive cardiomyopathy secondary to amyloid infiltration. However, these manifestations evolved progressively and were initially attributed to the primary psychiatric diagnosis and medication-related adverse effects.

### 3.2 Why might systemic amyloidosis be associated with depressive symptoms?

Although depressive symptoms have been reported in some patients with systemic amyloidosis (**Table 4**), an epidemiological study has also shown that 48.6% of patients with ATTR-type cardiac amyloidosis reported clinically significant depression or anxiety, or both (11), the mechanisms underlying this association remain insufficiently understood. Mechanistic insights into amyloid-related cognitive and affective disturbances have been primarily derived from Alzheimer’s disease and cerebral amyloid angiopathy (CAA), where vascular and parenchymal amyloid deposition has been associated with disrupted cognitive and emotional processing. Proposed pathways in these conditions include chronic inflammatory responses, oxidative stress, microvascular injury, white matter disruption, and blood–brain barrier dysfunction (12–14). However, the extent to which these mechanisms can be directly extrapolated to systemic amyloidosis remains uncertain. Emerging evidence from hereditary transthyretin amyloidosis and other systemic forms suggests that multisystem involvement, including vascular dysfunction, autonomic impairment, and systemic inflammatory burden, may be associated with neuropsychiatric manifestations. These effects are likely to reflect complex interactions between peripheral organ dysfunction and central nervous system regulation, rather than direct amyloid deposition within the brain (15, 16). In addition, endocrine and metabolic factors, such as thyroid dysfunction, may further contribute to fatigue, psychomotor slowing, and depressive symptoms in affected patients (17). Taken together, current evidence suggests that depressive symptoms in systemic amyloidosis may reflect a multifactorial process involving systemic disease burden, neurovascular regulation, and psychological response to chronic illness. However, these mechanisms remain largely hypothetical and require further investigation in larger studies.

**Table 4:** Reported amyloidosis cases presenting with affective or psychiatric symptoms.

Ref.	Author (Year)	Main somatic symptoms	Affective/psychiatric symptoms	Pathological findings	Amyloid type
(23)	Janot et al. (2010)	Chronic/subacute ileus after Whipple procedure, with lower abdominal pain, nausea, recurrent vomiting, and constipation/obstipation; intraoperatively, diffuse small-bowel adhesions and a “sugar-icing-like” coating causing severely impaired motility.	Severe depression in 1 patient, accompanied by cachexia and weakness.	Marked amyloid deposition in the serosa and subserosa of the small intestine; Congo red positive; immunohistochemistry confirmed AA amyloid.	AA amyloidosis
(24)	Fonnesu et al. (2009)	Chronic diarrhea, weight loss, dysphagia, xerostomia, xerophthalmia, and chronic gastritis, with electrocardiographic abnormalities and early restrictive cardiomyopathy.	Depression/anxious-depressive state.	Gastric/duodenal biopsy showed eosinophilic deposits in the mucosa, submucosa, and vessel walls, with apple-green birefringence on Congo red staining; urinary Bence Jones λ chains were present.	AL amyloidosis
(25)	Douglass et al. (2007)	Severe recurrent headaches often associated with focal neurological deficits, progressive ataxia, and later peripheral neuropathy; proteinuria and left ventricular wall thickening were also present.	Depression, personality change, and dementia/cognitive decline.	Endomyocardial biopsy confirmed transthyretin amyloid deposition; brain MRI suggested diffuse leptomeningeal amyloidosis; genetic testing identified the TTR Gly53Ala variant.	Hereditary transthyretin amyloidosis (ATTRv)
(26)	Knezevic et al. (2025)	Limb weakness, dizziness, vomiting, marked weight loss, cachexia, hypotension, tachycardia, sensory-motor-autonomic polyneuropathy, with renal and cardiac involvement.	Depressive symptoms, social withdrawal, loss of interest, low energy, and cognitive decline.	No positive tissue pathology was reported; diagnosis was established by ATTR-specific testing and genetic confirmation of a heterozygous TTR p.(Phe84Ser) pathogenic variant.	Hereditary transthyretin amyloidosis (ATTRv)
(27)	Brett et al. (1999)	Headache, recurrent subarachnoid hemorrhage, hearing loss, gait ataxia, urinary frequency/retention, sicca symptoms, constipation, orthostatic hypotension, peripheral neuropathy, cardiac involvement, vitreous opacities, and fluctuating consciousness.	Severe depression with fluctuating confusion/impaired consciousness.	Meningeal biopsy confirmed extensive amyloid deposition; Congo red positive, immunostaining consistent with TTR amyloid; genetic testing identified the TTR Leu12Pro variant.	Hereditary transthyretin amyloidosis (ATTRv)
(28)	Spaar et al. (1981)	Progressive visual loss, paracentral scotoma progressing to hemianopia, headache, vertigo, visual hallucinations, and focal mass-effect symptoms.	Recurrent depressive episodes.	Surgical pathology demonstrated cerebral amyloidoma; Congo red birefringence, thioflavine positivity, PAS positivity, predominant IgM immunofluorescence, and ultrastructural demonstration of typical non-branching 10-nm fibrils.	Localized cerebral amyloidoma/focal primary amyloid deposition

Janot et al. reported a three-patient case series; the table summarizes the case series at the article level.

### 3.3 Diagnostic overlap at the psychosomatic interface

The challenges encountered in this case align with existing evidence indicating significant diagnostic delays in cardiac amyloidosis. Even in patients with established heart failure, CA remains difficult to recognize. Reported mean and median diagnostic delays can span several years, with misdiagnosis rates reaching up to 50%, particularly pronounced in ATTR-CA cases. Patients often consult multiple healthcare providers before receiving an accurate diagnosis (18).

The primary drivers of this delay lie in the variability and non-specificity of early manifestations. Symptoms such as fatigue, reduced exercise tolerance, autonomic instability, and weight loss significantly overlap with those commonly seen in primary mood disorders. This phenotypic overlap increases the likelihood of early physiological abnormalities being misinterpreted within other diagnostic frameworks, thereby obscuring the progression of systemic pathology.

Cognitive factors may further exacerbate this susceptibility. When depressive symptoms emerge during the prodromal phase of medical conditions, anchoring bias may influence subsequent interpretations of new findings (19). A systematic review indicates that depression is the most frequently identified affective prodromal symptom in medical diseases, reported in Cushing’s syndrome, hypothyroidism, hyperparathyroidism, pancreatic and lung cancer, myocardial infarction, Wilson’s disease, and AIDS (20). Whilst these associations highlight the complex bidirectional relationship between mental and somatic disturbances, they simultaneously create opportunities for cognitive biases to arise. Once somatic symptoms are reattributed to an established psychiatric diagnosis, this impedes further investigation into their origin (2), potentially leading to misdiagnosis and missed diagnosis.

Beyond individual cognitive biases, systemic factors exacerbate diagnostic concealment. Limited interdisciplinary expertise, social stigmatization of mental illness, challenging-to-categories heterogeneous clinical presentations, and treatment strategies prioritizing symptom relief over etiological clarification collectively diminish patients’ access to more precise care within psychiatric settings compared to the general population (21, 22).

### 3.4 Clinical implications for psychiatric and multidisciplinary practice

While cardiac amyloidosis itself presents diagnostic challenges, this case offers several practical lessons for psychiatric and multidisciplinary care. When meeting diversity clinical features, clinicians should maintain heightened vigilance for somatic pathologies when affective symptoms are mild, atypical, or insufficient to explain the full clinical picture. Caution is warranted with new or persistent physiological abnormalities, especially signs suggestive of autonomic dysfunction (such as persistent orthostatic hypotension).

It is noteworthy that early clinical decisions in this case were made under conditions of incomplete and fragmented information. Treatment occurred across multiple institutions, and longitudinal data were not fully integrated. Consequently, each diagnostic step was based on the most prominent features at specific time points rather than comprehensive assessment of the disease course.

Ultimately, this case underscores the iterative, dynamic, and probabilistic nature of diagnostic reasoning. Effective clinical practice necessitates continually revising hypotheses as new data emerges, particularly at the mind–body interface where symptom overlap is common and cognitive biases may inadvertently narrow interpretative frameworks. In this context, the incorporation of standardized psychiatric rating scales (e.g., Hamilton Depression Rating Scale [HAM-D], Montgomery–Asberg Depression Rating Scale [MADRS], Patient Health Questionnaire-9 [PHQ-9], and Clinical Global Impression [CGI]) or other objective measures of symptom severity and treatment response may further enhance diagnostic precision and longitudinal assessment, particularly when differentiating primary psychiatric disorders from affective symptoms secondary to systemic disease. For clinicians, particularly those working at the psychiatric–somatic interface, the presence of affective symptoms should not lessen vigilance for an underlying medical disorder. When emotional symptoms are atypical, insufficient to explain the full clinical picture, or accompanied by persistent physiological abnormalities, further investigation for somatic causes should be undertaken. At the same time, physical comorbidities should be actively identified and addressed through multidisciplinary comorbidity management rather than being considered secondary to the psychiatric presentation alone.

In the present case, the patient’s evolving clinical features can be interpreted within the systemic–brain framework outlined above, although direct mechanistic confirmation is not available. While the initial diagnosis of a major depressive episode was clinically understandable on the basis of low mood, anhedonia, sleep disturbance, appetite loss, and marked weight loss, the subsequent emergence of persistent orthostatic hypotension, recurrent syncope, progressive dysphagia, and biomarker evidence of cardiac stress suggested a broader multisystem process that could not be fully explained by a primary psychiatric disorder alone. These features are consistent with the possibility of autonomic dysfunction, systemic organ involvement, and hemodynamic instability associated with amyloid disease. In this context, the patient’s affective symptoms may have reflected not only the psychological burden of chronic illness but also underlying biological processes potentially involving inflammation, oxidative stress, neurovascular dysfunction, and systemic–brain interactions.

From a clinical management perspective, in patients with systemic amyloidosis presenting with orthostatic hypotension, repeated supine–standing blood pressure measurements may be useful even after apparent hemodynamic stabilization, as they can help assess persistent autonomic dysfunction and guide strategies aimed at improving fatigue and mood symptoms related to systemic hypoperfusion. In addition, cognitive assessment using standardized screening tools, such as the Mini-Mental State Examination (MMSE) or Montreal Cognitive Assessment (MoCA), may be helpful in excluding potential central nervous system involvement and in better characterize the extent of systemic–brain interaction.

During the subsequent clinical management of this case, the limited response to antidepressant treatment and the subsequent improvement in mood symptoms following stabilization of the underlying somatic condition further support the possibility that his depressive presentation was, at least in part, linked to the pathophysiology of systemic amyloidosis rather than representing an entirely independent psychiatric disorder. Accordingly, this case does not establish causality, but it provides a clinically plausible model linking systemic amyloid disease, multisystem dysfunction, and prominent depressive symptoms.

Recent studies have indicated that systemic amyloidosis is being recognized more frequently in contemporary practice, especially with increased awareness and advances in non-invasive diagnostic strategies. Therefore, systemic amyloidosis should be included more readily in the diagnostic and differential diagnostic work-up of patients presenting with depressive symptoms alongside unexplained multisystem manifestations.

## 4 Conclusion

This case highlights diagnostic overshadowing phenomena at the intersection of psychiatry and somatic medicine. Cardiac amyloidosis may present with non-specific multisystem symptoms overlapping with depressive symptoms, increasing risks of misattribution and delayed recognition. Under conditions of incomplete information and fragmented care, anchoring bias may constrain diagnostic reasoning and impede timely reassessment.

For clinicians, particularly those practicing psychiatry, sustained vigilance regarding evolving physiological abnormalities—such as persistent orthostatic hypotension or unexplained systemic symptoms—remains paramount. Diagnostic reasoning should remain dynamically iterative, with careful re-evaluation of alternative etiologies as new evidence emerges. Enhancing interdisciplinary awareness and mitigating cognitive biases may help reduce diagnostic delays and improve outcomes for patients presenting with complex psychosomatic symptoms.

Additionally, depressive symptoms in systemic amyloidosis may not solely reflect a psychological response to chronic illness but could also be associated with complex systemic and neurobiological interactions. However, the underlying mechanisms remain incompletely understood. Greater clinical awareness and multidisciplinary evaluation are essential to reduce diagnostic delay and improve patient outcomes.

## Data Availability

The original contributions presented in the study are included in the article/supplementary material. Further inquiries can be directed to the corresponding authors.
